# Sustainable Production and Antioxidant Activity of Bacterial Xanthan Gum

**DOI:** 10.3390/molecules30132734

**Published:** 2025-06-25

**Authors:** Ilona Jonuškienė, Erika Davicijonaitė, Monika Vaškevičiūtė, Ihsan Kala, Rima Stankevičienė, Kristina Kantminienė, Ingrida Tumosienė

**Affiliations:** 1 Department of Organic Chemistry, Kaunas University of Technology, Radvilėnų Pl. 19, LT-50254 Kaunas, Lithuania; erikutedavi@gmail.com (E.D.); monika.vaskeviciute99@gmail.com (M.V.); ihsan.kala13@gmail.com (I.K.); rima.stankeviciene@ktu.lt (R.S.); ingrida.tumosiene@ktu.lt (I.T.); 2 Bioprocess Research Centre, Kaunas University of Technology, Radvilėnų Pl. 19, LT-50254 Kaunas, Lithuania; 3 Department of Physical and Inorganic Chemistry, Kaunas University of Technology, Radvilėnų Pl. 19, LT-50254 Kaunas, Lithuania; kristina.kantminiene@ktu.lt

**Keywords:** biopolymers, carbon and nitrogen sources, renewable resources, antioxidant activity

## Abstract

One of the world’s most sustainable solutions is to replace fossil-based polymers with biopolymers. The production of xanthan gum can be optimized using various renewable and cost-effective raw materials, which is a key focus in industrial biotechnology. Xanthan gum is a bioengineered thickening, stabilizing, and emulsifying agent. It has unique properties for use in many industries (food, biotechnology, petrochemicals, agricultural, cosmetics, wastewater treatment) and medical applications. It is tasteless, environmentally safe, non-toxic, and biodegradable. The biotechnological production of xanthan gum depends on several factors: bacterial strain development, culture medium preparation, carbon sources, fermentation parameters and modes, pH, temperature, recovery, purification, and quality control regulations. Bio-innovative strategies have been developed to optimize the production of xanthan gum. A variety of carbon and nitrogen sources, as well as alternative renewable sources, have been used in the production of xanthan gum. The aim of the present study was to optimize the xanthan gum yield using *Xanthomonas campestris* bacteria and different carbon (D-glucose, D-sorbitol, lactose, sucrose, D-mannitol, D-fructose, erythritol, coconut palm sugar, L-arabinose, unrefined cane sugar), various nitrogen (bacterial peptone, casein peptone, L-glutamic acid, L-arginine, L-methionine, L-tryptophan, malt extract, meat extract, L-phenylalanine, soy peptone) and alternative carbon (orange peels, tangerine peels, lemon peels, avocado peels, melon peels, apple peels, cellulose, xylose, xylitol) sources. The xanthan gum samples were analyzed using antioxidant methods. Our study showed that using L-glutamic acid as the carbon source for 72 h of bacterial fermentation of *Xanthomonas campestris* resulted in the highest xanthan gum yield: 32.34 g/L. However, using renewable resources, we achieved a very high concentration of xanthan gum in just 24 h of fermentation. According to the reducing power and DPPH methods, the highest antioxidant activities were measured for xanthan gum whose biosynthesis was based on renewable resources. Xanthan gum structures have been verified by FT-IR and ^1^H NMR analysis. The sustainable biotechnology study has the advantage of increasing the sustainable production of xanthan gum by using renewable alternative resources compared to other production processes. Xanthan gum continues to be a valuable biopolymer with a wide range of industrial applications while promoting environmentally friendly production practices.

## 1. Introduction

The replacement of fossil-based polymers with biopolymers is one of the most sustainable solutions. Biopolymers are environmentally friendly; they are produced from renewable resources, and their degradation does not increase the concentration of CO_2_ in the environment. In recent decades, interest in microbial production of xanthan gum has increased due to the growing demand for high-quality bioproducts in various industries. Based on applications, the market for xanthan gum is divided into oil and gas, food and beverages, cosmetics, pharma, and others [1]. The food and beverage segment is expected to reach a volume of over 160 kilotons by 2030. Commercial xanthan gum is a thickening agent, a tasteless, dry, white to cream-colored powder used in the production of jams, puddings, sauces, canned and frozen foods, and beverages [1,2]. The valuable commercial bacterial heteropolysaccharide xanthan gum is produced by several strains of *Xanthomonas* bacteria, among which *Xanthomonas campestris* is the most widely used strain [3]. Xanthan gum is environmentally friendly, biocompatible, and biodegradable [4]. Its biological and pharmacological activities include antibacterial, antioxidant, and anticancer ones. Xanthan gum exhibits pseudoplastic properties, high viscosity, solubility, pH, and temperature stability [5].

The biotechnological production of xanthan gum depends on the choice of bacterial strain development, carbon and nitrogen sources, composition of the culture medium, fermentation parameters and modes, cell removal, isolation and recovery, purification of the final biopolymer, and regulatory approval of quality control.

The biotechnological production of xanthan gum involves the following steps: preparation of *Xanthomonas campestris* inoculum and carbon sources, aerobic and submerged fermentation, cell removal, recovery, and purification of the final product [2,6,7]. The production and properties of xanthan gum have been found to be influenced by several factors: physiological and genetic state of bacterial cells [8,9], culture medium [10,11,12,13], nutrient source [14,15,16,17,18,19,20], temperature [21,22], pH [23], fermentation time [24], bioreactor type [25], fermentation mode (batch or continuous), airflow [26,27], impeller type [28,29,30], oxygenation, upstream and downstream processes.

Various carbon sources have been reported to produce xanthan gum. In general, the concentration of the carbon source affects the conversion yield of sugar to polysaccharide [31]. The substrate is taken up from the culture medium and converted into intermediate nucleotide derivatives, which are then further converted into monomer units, polymerized into polysaccharide chains, and exported to the extracellular environment [32].

A backbone of β-(1,4)-linked D-glucose units with a trisaccharide side chain on alternating glucose residues forms the primary structure of xanthan gum. The side chain of this trisaccharide is composed of two mannose units, which are separated by a glucuronic acid. Xanthan-derived oligosaccharides are known to have antioxidant, antibacterial, and eliciting activities [33].

Due to its interactions with cations (i.e., Na^+^, K^+^, Ca^2+^, and Mg^2+^), xanthan gum can be considered an anionic polyelectrolyte, which is bound to acidic residues in varying proportions [34]. However, there are still several challenges to overcome to increase the sustainable industrial production and applications of xanthan gum. One of the disadvantages of xanthan gum is the cost of the carbon sources used in its production, the complexity of the extraction and production technologies, and, therefore, the higher cost of the resulting bioengineering product.

Xanthan gum is produced industrially using sucrose and glucose as carbon sources, but rising prices are forcing the search for alternative renewable feedstocks that can be adapted for biotechnological production of the biopolymer. The cost of carbon accounts for a third of the bioproduction of xanthan gum. Therefore, alternatives are being sought to reduce costs and increase sustainability. The use of agricultural and industrial waste as carbon sources is an important alternative to reduce production costs. However, the successful conversion of some of these nutrient sources into xanthan gum remains a challenge [35].

The aim of the present study was threefold: to optimize the production conditions of xanthan gum using the bacterium *Xanthomonas campestris* (including the use of different carbon, nitrogen and renewable resources, as well as fermentation time); to increase the yield of xanthan gum; and to evaluate the antioxidant activity of the obtained xanthan gum using the FRAP, reducing power and DPPH methods. FT-IR and ^1^H NMR analyses were also performed.

Here, we discuss the main challenges in understanding the microbial sustainable production of xanthan gum and the current strategies to increase its yield by combining three targets (alternative carbon and nitrogen sources, fermentation time, and antioxidant activity) in biopolymer biosynthesis.

## 2. Results

*Xanthomonas campestris* inoculum requires carbon sources, micronutrients, and macronutrients for xanthan gum biosynthesis. The concentration of the carbon source affects the xanthan gum yield; a concentration of 2 ± 4% is preferred [5]. The culture medium provides nutrients for microbial growth. It plays a crucial role in the molecular structure, biosynthesis, recovery, and production yield of xanthan gum. Glucose and sucrose are commonly used and continue to be important sources of carbon for both yield and quality [34]. Therefore, costs can be reduced, and a higher quality biopolymer can be produced by optimizing the type and concentration of nutrients (mainly the carbon source) [35]. To reduce production costs, many researchers have started using alternative carbon sources to produce xanthan gum. Examples of these alternative sources are apple juice residues, date juice palm, sugar beet molasses, sugarcane molasses, crude glycerol, kitchen waste hydrolysate, orange peels, jackfruit seed powder, and cheese whey [5,36]. The minimum nutritional requirements for the biosynthesis of xanthan gum are carbon and nitrogen sources and micronutrients [36].

Bioproduction of xanthan gum using various carbon sources. In this study, the best combination of xanthan gum yield was obtained when the following parameters were applied: pH 7.0, 37 °C, and 150 rpm during fermentation for 24–72 h.

**Figure 1:**
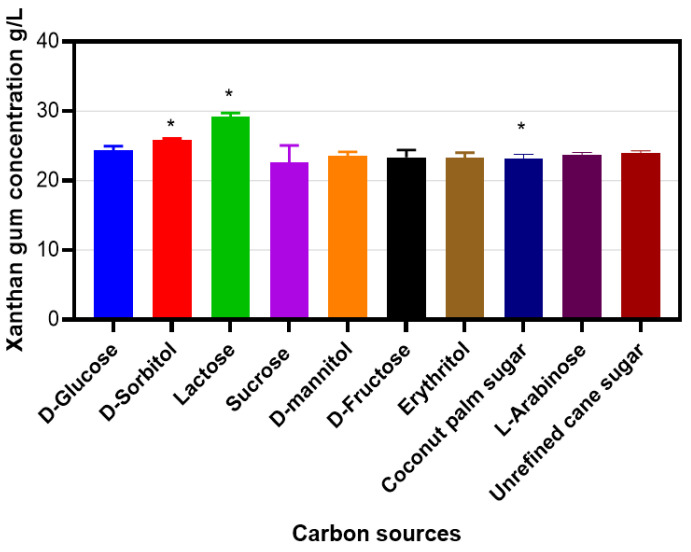
Xanthan gum concentration after 24 h of fermentation using carbon resources. The error bars show the mean ± SD of three experiments. Asterisks show statistically significant comparisons between D-glucose and alternative carbon sources, which were tested with an unpaired two-tailed *t*-test, with a significance level set at * *p* < 0.1.

**Figure 2:**
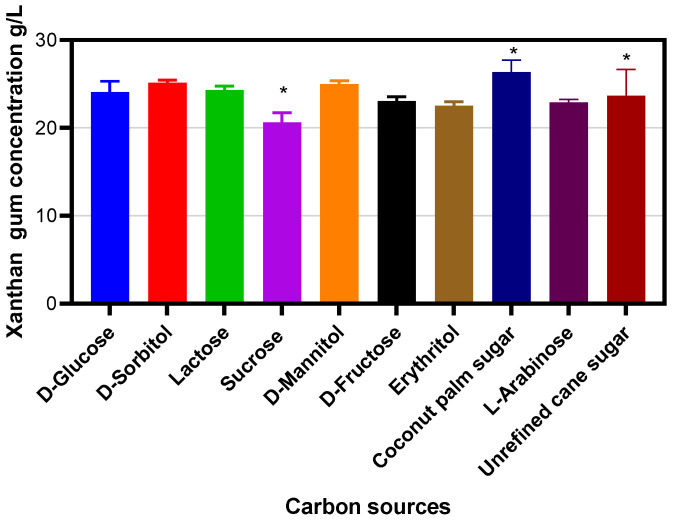
Xanthan gum concentration after 48 h of fermentation using carbon resources. The error bars show the mean ± SD of three experiments. Asterisks show statistically significant comparisons between D-glucose and alternative carbon sources, which were tested with an unpaired two-tailed *t*-test, with a significance level set at * *p* < 0.1.

**Figure 3:**
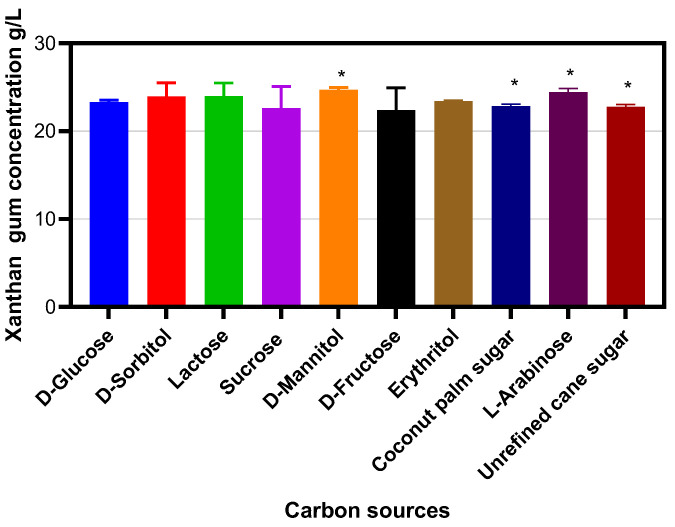
Xanthan gum concentration after 72 h of fermentation using carbon resources. The error bars show the mean ± SD of three experiments. Asterisks show statistically significant comparisons between D-glucose and alternative carbon sources, which were tested with an unpaired two-tailed *t*-test, with a significance level set at * *p* < 0.1.

After 24–72 h of fermentation, the concentration of xanthan gum obtained by biosynthesis using different carbon sources was determined as shown in Figure 1, Figure 2 and Figure 3.

The biosynthetic pathway to produce xanthan gum involves several steps: (1) assembly of pentasaccharide subunits attached to an inner membrane polyprenol phosphate carrier, (2) addition of acetyl and pyruvate groups, (3) polymerization of the pentasaccharide repeating units and secretion of xanthan gum [37]. It is useful to consider the chemical structure of the carbon source used when analyzing the results of the study.

It was found (Figure 1) that after 24 h of fermentation, the highest concentrations of xanthan gum were obtained with the addition of lactose (29.19 g/L) compared to D-glucose (24.37 g/L). Previous research by scientists showed that the highest concentration (28 g/L) was achieved using hydrolyzed whey lactose supplemented with sucrose and diammonium phosphate. Xanthan gum can be successfully produced using lactose as a substrate [38]. According to the literature data, the highest xanthan gum yield was 14.74 g/L with glucose [39,40]. Also, previous experiments showed that sucrose and glucose produced the best results in terms of the quality of xanthan gum, which is characterized by high viscosity and high molecular weight [41].

Monosaccharide polyols, including erythritol, xylitol, and D-sorbitol, are low molecular weight carbohydrates that are commonly used as sweeteners in pharmaceutical and food applications [42]. Erythritol is used as a natural sweetener in the food and pharmaceutical industries. It has approximately 70% of the relative sweetness of sucrose and was approved by the FDA in 2001 as a low-calorie sweetener [43]. Erythritol has been shown to have oral health benefits by inhibiting the growth of oral bacteria, in addition to its sweetening properties [44].

When the results of the study were analyzed, it was found that the highest concentrations of xanthan gum (Figure 2) were obtained using coconut palm flower sugar (26.37 g/L), with D-sorbitol (25.14 g/L) and D-mannitol (24.98 g/L) as carbon sources, compared to D-glucose (24.06 g/L) after 48 h of fermentation.

After 72 h of fermentation, the yield of xanthan gum was using D-glucose—23.32 g/L. When analyzing the results of the study, it was found that the highest concentrations of xanthan gum (Figure 3) were obtained using D-mannitol (24.72 g/L) and L-arabinose (24.45 g/L) as carbon sources compared to D-glucose (23.32 g/L) after 72 h of fermentation.

**Figure 4:**
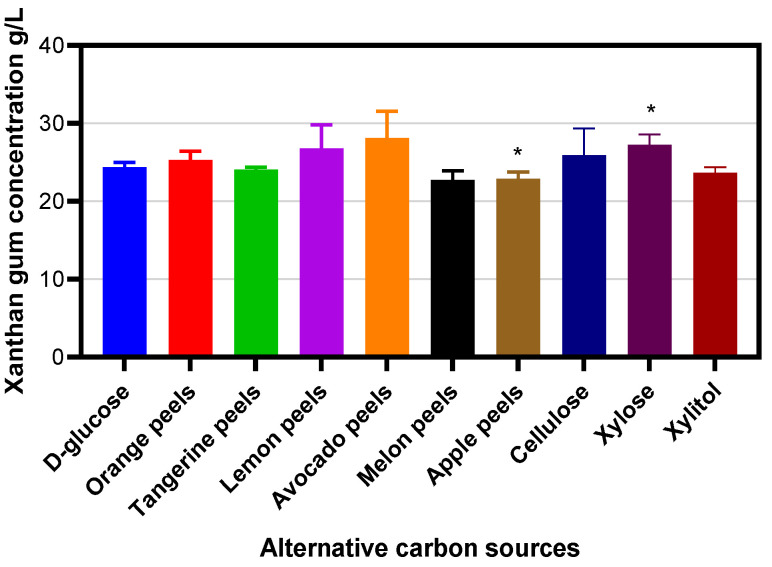
Xanthan gum concentration after 24 h of fermentation using alternative carbon resources. The error bars show the mean ± SD of three experiments. Asterisks show statistically significant comparisons between D-glucose and alternative carbon sources, which were tested with an unpaired two-tailed *t*-test, with a significance level set at * *p* < 0.1.

**Figure 5:**
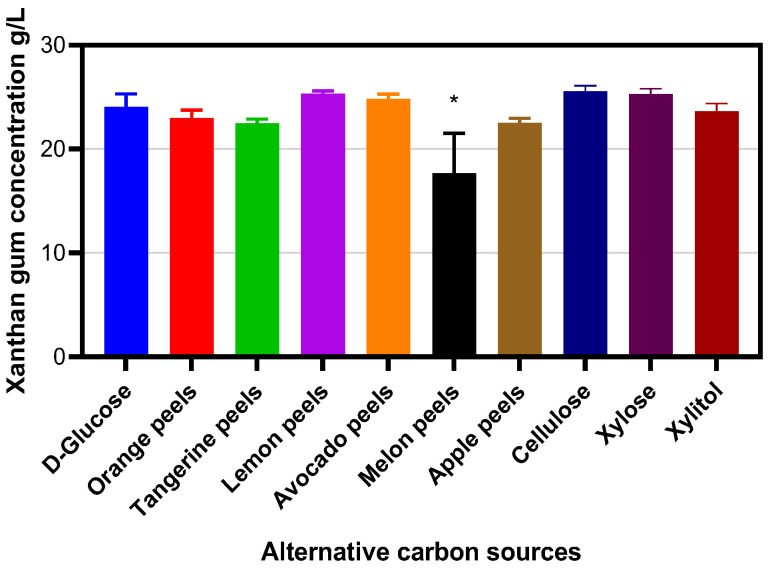
Xanthan gum concentration after 48 h of fermentation using alternative carbon resources. The error bars show the mean ± SD of three experiments. Asterisks show statistically significant comparisons between D-glucose and alternative carbon sources, which were tested with an unpaired two-tailed *t*-test, with a significance level set at * *p* < 0.1.

**Figure 6:**
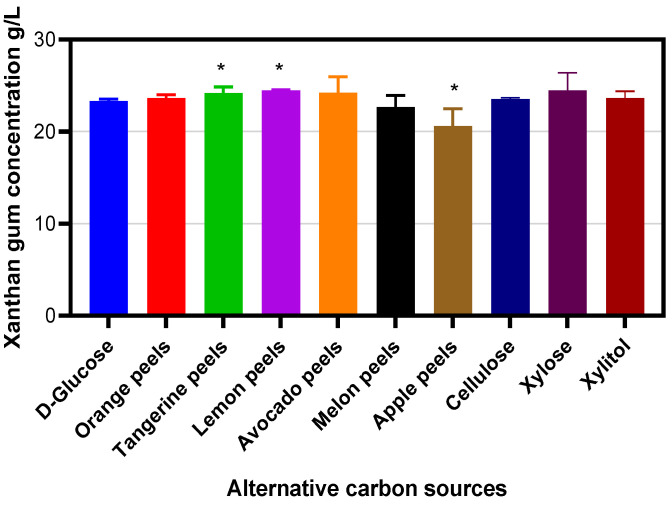
Xanthan gum concentration after 72 h of fermentation. The error bars show the mean ± SD of three experiments. Asterisks show statistically significant comparisons between D-glucose and alternative carbon sources, which were tested with an unpaired two-tailed *t*-test, with a significance level set at * *p* < 0.1.

The influence of alternative sources and renewable resources on the production of xanthan gum is described as follows. Previous research has shown that enhanced sustainable bioproduction of xanthan gum can be achieved by utilizing orange peels. Furthermore, optimized treatment in a 15 L bioreactor enabled the highest xanthan production of 30.19 g/L from orange peels to be achieved [45]. After 24–72 h of fermentation, the concentration of xanthan gum obtained by biosynthesis using different alternative carbon sources was determined as shown in Figure 4, Figure 5 and Figure 6.

After 24 h of fermentation, when analyzing the results of the study, it was found that the highest concentrations of xanthan gum (Figure 4) were obtained using avocado peels (28.09 g/L), xylose (27.20 g/L), peels of lemon (26.77 g/L) as carbon sources compared to D-glucose (24.37 g/L) after 24 h of fermentation.

After 48 h of fermentation, when analyzing the results of the study, it was found that the highest concentrations of xanthan gum (Figure 5) were obtained using cellulose (25.56 g/L), peels of lemon (25.34 g/L), xylose (25.29 g/L), as carbon sources compared to D-glucose (24.06 g/L) after 48 h of fermentation.

After 72 h of fermentation, when analyzing the results of the study, it was found that the highest concentrations of xanthan gum (Figure 6) were obtained using xylose (24.49 g/L), lemon peels (24.48 g/L), and tangerine peels (24.20 g/L) as carbon sources compared to D-glucose (23.31 g/L) after 72 h of fermentation.

Previous research has shown that sour lemon peel can be used to make a biodegradable film. The physicochemical properties of this film can be modified by adding xanthan gum and TiO_2_–Ag nanoparticles [46].

**Figure 7:**
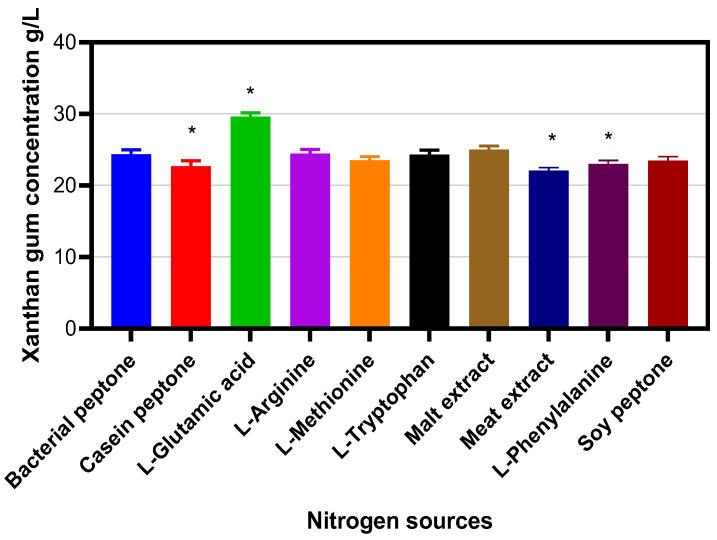
Xanthan gum concentration after 24 h of fermentation using nitrogen resources. The error bars show the mean ± SD of three experiments. Asterisks show statistically significant comparisons between bacterial peptone and alternative nitrogen sources, which were tested with an unpaired two-tailed *t*-test, with a significance level set at * *p* < 0.1.

**Figure 8:**
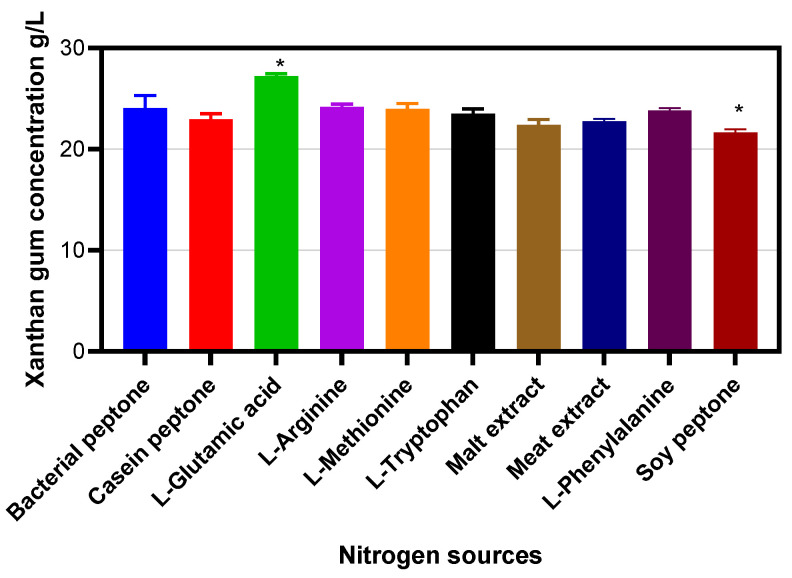
Xanthan gum concentration after 48 h of fermentation using nitrogen resources. The error bars show the mean ± SD of three experiments. Asterisks show statistically significant comparisons between bacterial peptone and alternative nitrogen sources, which were tested with an unpaired two-tailed *t*-test, with a significance level set at * *p* < 0.1.

**Figure 9:**
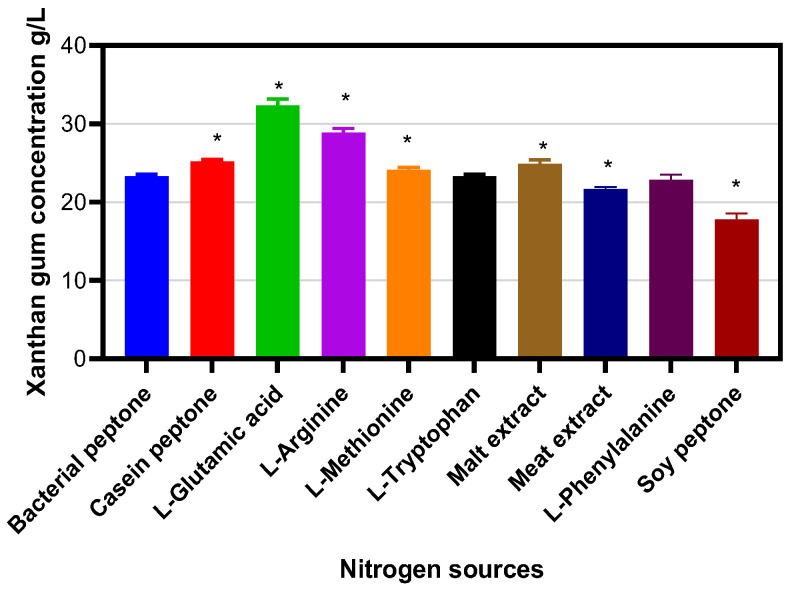
Xanthan gum concentration after 72 h of fermentation using nitrogen resources. The error bars show the mean ± SD of three experiments. Asterisks show statistically significant comparisons between bacterial peptone and alternative nitrogen sources, which were tested with an unpaired two-tailed *t*-test, with a significance level set at * *p* < 0.1.

The influence of nitrogen sources on xanthan gum production. Previous research has shown that amino acids such as cysteine, alanine, and histidine produce good yields of xanthan gum [38]. The concentration of xanthan gum obtained by biosynthesis using different alternative nitrogen sources was determined after 24–72 h of fermentation, as shown in Figure 7, Figure 8 and Figure 9.

After 24 h of fermentation, when analyzing the results of the study, it was found that the highest concentrations of xanthan gum (Figure 7) were obtained using L-glutamic acid (29.61 g/L) and malt extract (24.99 g/L) as nitrogen sources compared to bacterial peptone (24.37 g/L) after 24 h of fermentation.

After 48 h of fermentation, when analyzing the results of the study, it was found that the highest concentrations of xanthan gum (Figure 8) were obtained using L-glutamic acid (27.21 g/L), L-arginine (24.16 g/L), L-phenylalanine (23.80 g/L) as nitrogen sources compared to bacterial peptone (24.06 g/L) after 48 h of fermentation.

After 72 h of fermentation, when analyzing the results of the study, it was found that the highest concentrations of xanthan gum (Figure 9) were obtained using L-glutamic acid (32.34 g/L), L-arginine (28.89 g/L), and casein protein (25.18 g/L) as nitrogen sources compared to bacterial peptone (23.31 g/L) after 72 h of fermentation.

Evaluation of the antioxidant activity of xanthan gum. The antioxidant activity of xanthan gum has attracted increasing attention as antioxidant health products have become popular in recent decades [47]. The biological activities of biopolymers are closely related to their structure, such as the composition of monosaccharides and the substitutions [48,49]. For example, pyruvate acid levels may reflect the association with the radical scavenging activity of xanthan oligosaccharides [50]. Furthermore, polyglucuronic oxidized xanthan and O-acetylated algal polysaccharides showed improvements in the hydroxyl radical scavenging activities [33,51,52].

**Figure 10:**
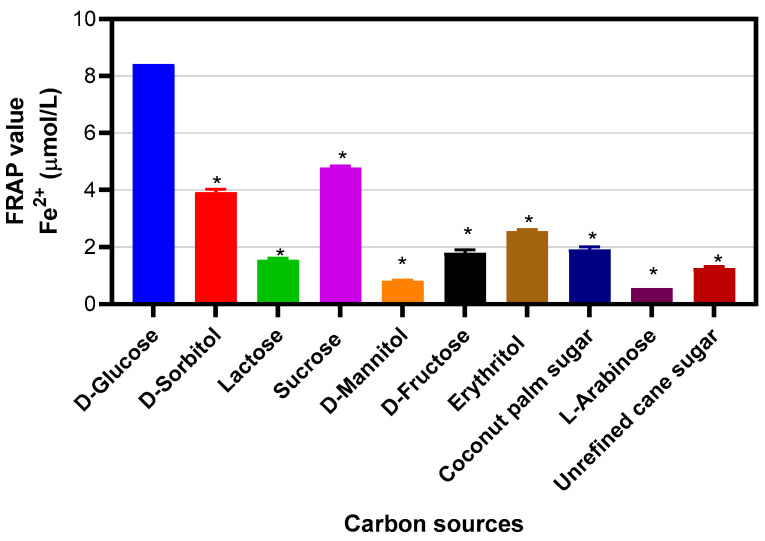
Antioxidant activity of extracted xanthan gum (from analyzed carbon sources) using the FRAP method. The error bars show the mean ± SD of three experiments. Asterisks show statistically significant comparisons between D-glucose and alternative carbon sources, which were tested with an unpaired two-tailed *t*-test, with a significance level set at * *p* < 0.01.

**Figure 11:**
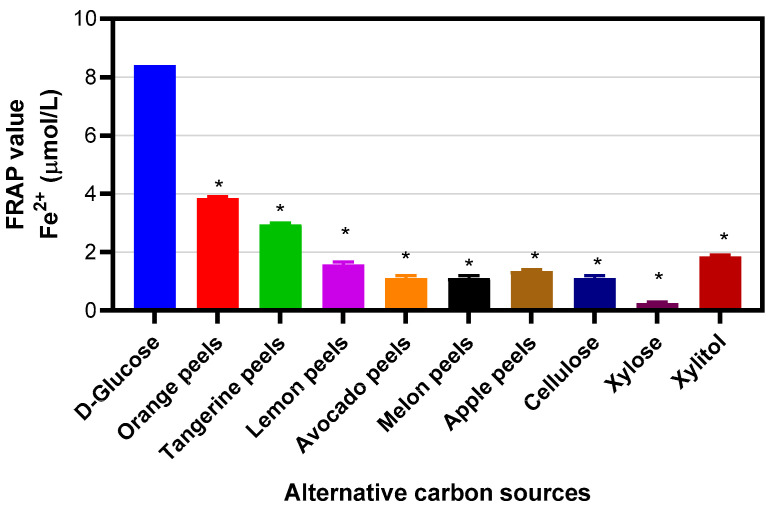
Antioxidant activity of extracted xanthan gum (from analyzed alternative carbon sources) using the FRAP method. The error bars show the mean ± SD of three experiments. Asterisks show statistically significant comparisons between D-glucose and alternative carbon sources, which were tested with an unpaired two-tailed *t*-test, with a significance level set at * *p* < 0.01.

**Figure 12:**
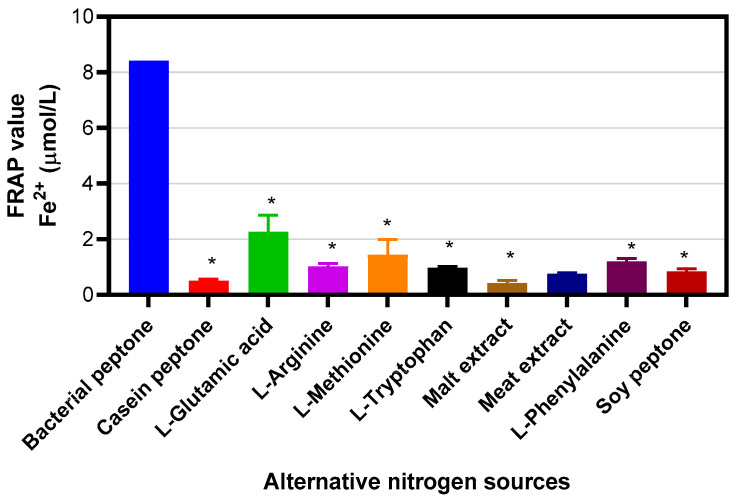
Antioxidant activity of extracted xanthan gum (from analyzed alternative nitrogen sources) using the FRAP method. The error bars show the mean ± SD of three experiments. Asterisks show statistically significant comparisons between bacterial peptone and alternative nitrogen sources, which were tested with an unpaired two-tailed *t*-test, with a significance level set at * *p* < 0.01.

**Figure 13:**
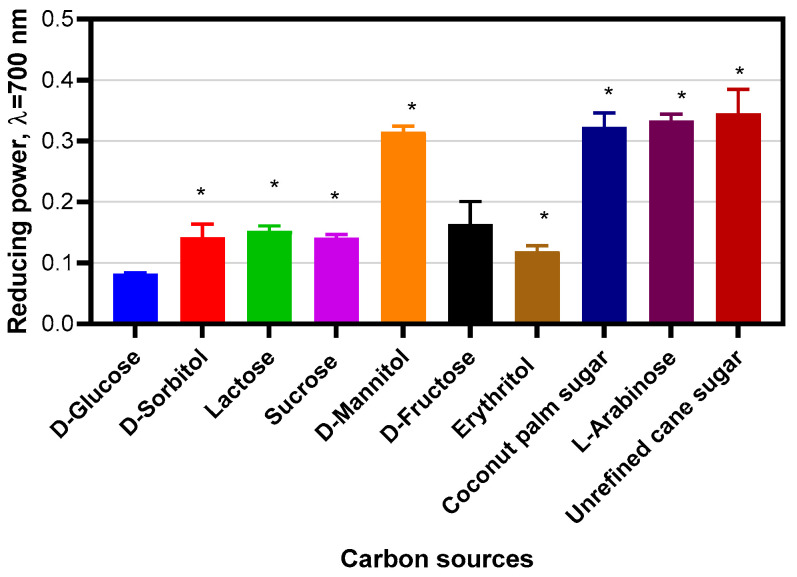
Antioxidant activity of extracted xanthan gum (from analyzed carbon sources) using the reducing power method. The error bars show the mean ± SD of three experiments. Asterisks show statistically significant comparisons between D-glucose and carbon sources, which were tested with an unpaired two-tailed *t*-test, with a significance level set at * *p* < 0.01.

**Figure 14:**
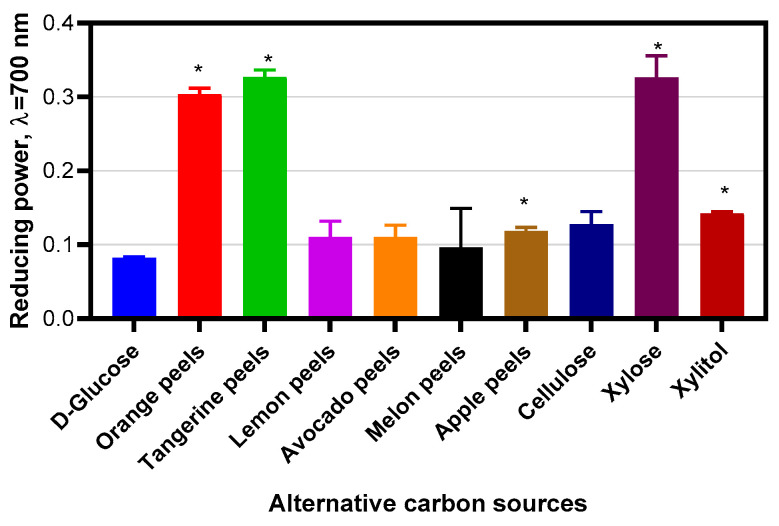
Antioxidant activity of extracted xanthan gum (from analyzed alternative carbon sources) using the reducing power method. The error bars show the mean ± SD of three experiments. Asterisks show statistically significant comparisons between D-glucose and alternative carbon sources, which were tested with an unpaired two-tailed *t*-test, with a significance level set at * *p* < 0.01.

**Figure 15:**
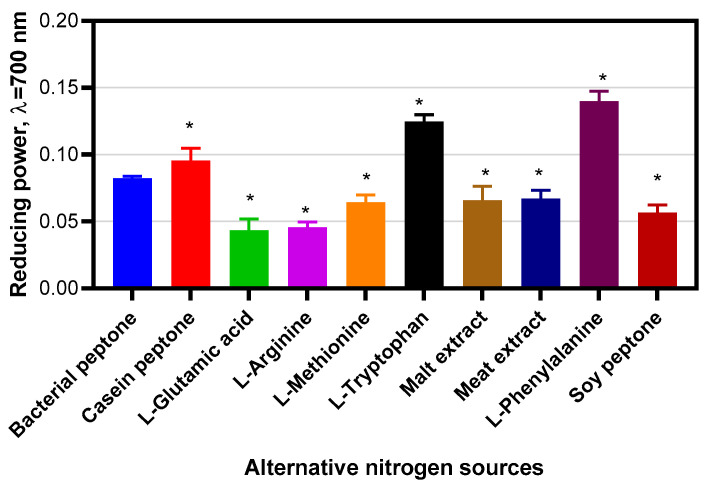
Antioxidant activity of extracted xanthan gum (from analyzed alternative nitrogen sources) using reducing power method. The error bars show the mean ± SD of three experiments. Asterisks show statistically significant comparisons between bacterial peptone and alternative nitrogen sources, which were tested with an unpaired two-tailed *t*-test, with a significance level set at * *p* < 0.01.

Antioxidant activity according to the FRAP and reducing power assays. Xanthan gum was synthesized using different carbon and nitrogen sources, and then a study of the reducing properties was conducted using the FRAP and reducing power methods, the results of which are shown in Figure 10, Figure 11, Figure 12, Figure 13, Figure 14 and Figure 15.

The highest antioxidant activity was measured for xanthan gum (Figure 10), whose biosynthesis was based on D-glucose (8.42 μmol/L). The results of the study showed that lower antioxidant activity of xanthan gum samples was obtained when other carbon sources were used, i.e., sucrose (4.82 μmol/L), D-sorbitol (3.95 μmol/L), and erythritol (2.55 μmol/L).

The highest antioxidant activity was measured for xanthan gum (Figure 11), whose biosynthesis was based on D-glucose (8.42 μmol/L). The results of the study showed that lower antioxidant activity of xanthan gum samples was obtained when other alternative carbon sources were used, i.e., orange peels (3.85 μmol/L), tangerine peels (2.95 μmol/L), and xylitol (1.85 μmol/L).

The highest antioxidant activity was measured for xanthan gum (Figure 12), whose biosynthesis was based on bacterial peptone (8.42 μmol/L). The results of the study showed that lower antioxidant activity of xanthan gum samples was obtained when other alternative nitrogen sources were used, i.e., L-glutamic acid (2.27 μmol/L), L-methionine (1.44 μmol/L), and L-phenylalanine (1.21 μmol/L).

The highest antioxidant activities were measured for xanthan gum (Figure 13), whose biosynthesis was based on unrefined cane sugar (0.35 o.u.), L-arabinose (0.33 o.u.), and coconut palm sugar (0.32 o.u.), in comparison with D-glucose (0.082 o.u.).

The highest antioxidant activities according to reducing power method and using renewable resources were measured for xanthan gum (Figure 15), whose biosynthesis was based on tangerine peels (0.33 o.u.), xylose (0.33 o.u.), orange peels (0.3 o.u.), in comparison with D-glucose (0.082 o.u.).

The highest antioxidant activities were measured for xanthan gum (Figure 14), whose biosynthesis was based on L-phenylalanine (0.14 o.u.), L-tryptophan (0.12 o.u.), casein peptone (0.1 o.u.), in comparison with bacterial peptone (0.082 o.u.).

Antioxidant activity according to the DPPH assay. Xanthan gum has a protective effect against the hydroxyl radical (*OH), which is the reactive oxygen species (ROS) that is most harmful to biological tissues [51]. The antioxidant capacity of the main components of xanthan gum (glucose and mannose) is very similar, although it is known that monosaccharide content plays an important role in the ability to scavenge hydroxyl radicals [52].

**Figure 16:**
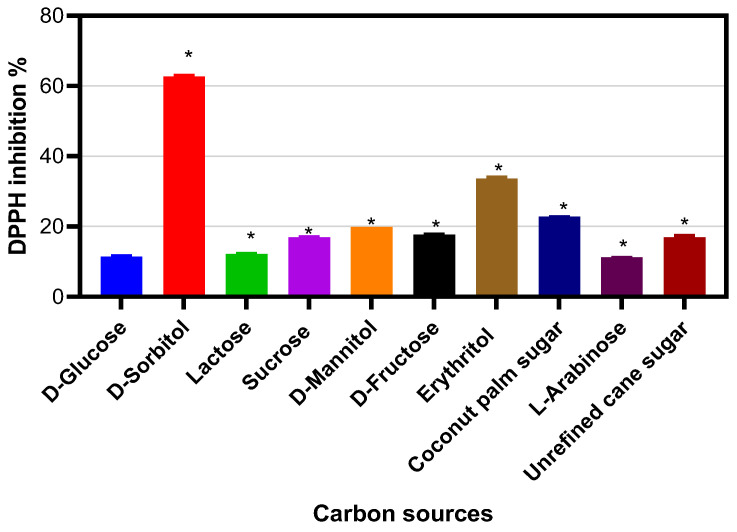
Antioxidant activity of xanthan gum according to the DPPH method (from analyzed carbon sources). The error bars show the mean ± SD of three experiments. Asterisks show statistically significant comparisons between D-glucose and alternative carbon sources, which were tested with unpaired two-tailed *t*-test, with a significance level set at * *p* < 0.01.

The antioxidant activity of xanthan gum samples obtained from different carbon sources was tested by DPPH assay. The results obtained are shown in Figure 16.

The highest antioxidant activity according to the DPPH was obtained for xanthan gum, for which a biosynthesis of D-sorbitol (62.67%) was used as the carbon source. The high antioxidant activities of xanthan gum were also obtained using erythritol supplement (33.75%) and coconut palm flower sugar (22.83%) compared to D-glucose (11.44%) (Figure 16).

**Figure 17:**
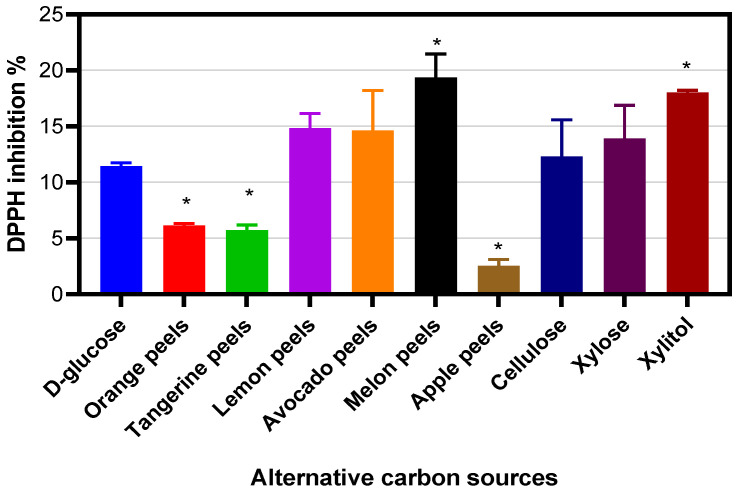
Antioxidant activity of xanthan gum according to the DPPH method (from analyzed alternative carbon sources). The error bars show the mean ± SD of three experiments. Asterisks show statistically significant comparisons between D-glucose and alternative carbon sources, which were tested with unpaired two-tailed *t*-test, with a significance level set at * *p* < 0.01.

The highest antioxidant activity according to the DPPH was obtained for xanthan gum, for which a biosynthesis of melon peels (19.36%) was used as the carbon source. The high antioxidant activities of xanthan gum were also obtained using lemon peels (14.83%) and peels of avocado (14.64%) compared to D-glucose (11.44%) (Figure 17).

**Figure 18:**
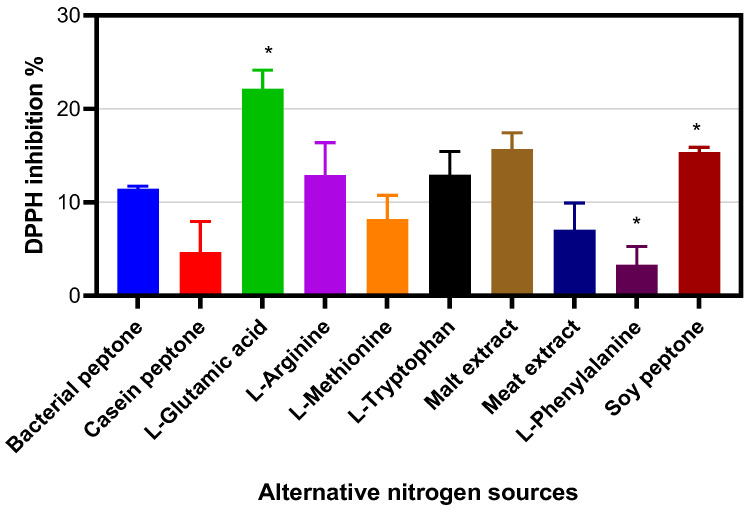
Antioxidant activity of extracted xanthan gum (from analyzed alternative nitrogen sources) using DPPH method. The error bars show the mean ± SD of three experiments. Asterisks show statistically significant comparisons between bacterial peptone and alternative nitrogen sources, which were tested with an unpaired two-tailed *t*-test, with a significance level set at * *p* < 0.01.

The highest antioxidant activity according to the DPPH was obtained for xanthan gum, for which a biosynthesis of L-glutamic acid (22.14%) was used as the carbon source. The high antioxidant activities of xanthan gum were also obtained using malt extract (15.68%) and soy peptone (15.38%) compared to bacterial peptone (7.96%) (Figure 18).

**Figure 19:**
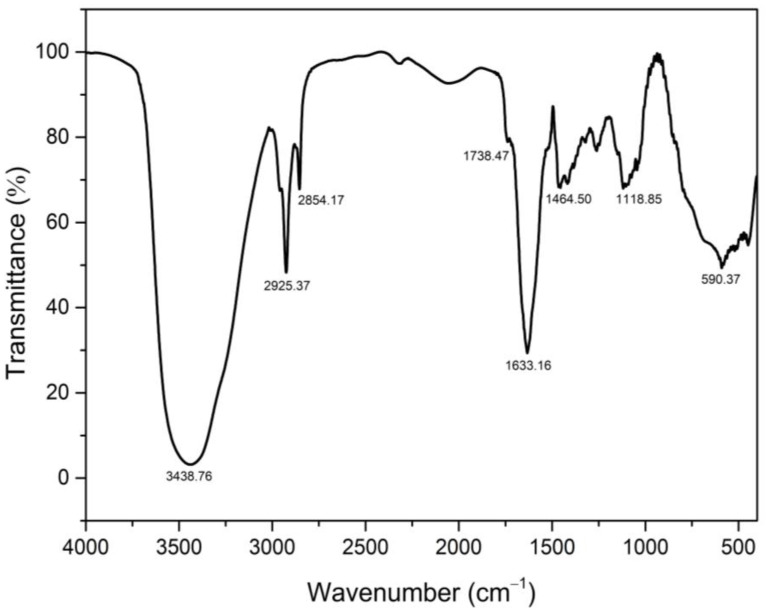
FT-IR spectra of biosynthesized xanthan gum using D-glucose as the carbon source.

**Figure 20:**
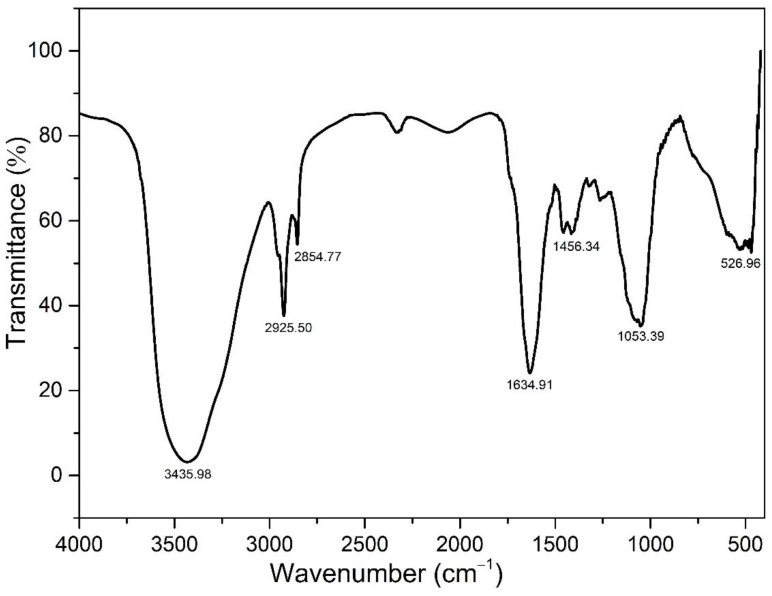
FT-IR spectra of biosynthesized xanthan gum using coconut palm sugar as the carbon source.

FT-IR spectra: The FT-IR spectrum was recorded for a sample of biosynthesized xanthan gum using D-glucose as the carbon source (Figure 19) and coconut palm sugar as the carbon source (Figure 20).

The broad absorption bands (Figure 19 and Figure 20) observed at approximately 3438.76–3435.98 cm^−1^ have been ascribed to O-H stretching, while those at 2925.37–2925.50 cm^−1^ are due to C-H stretching. The absorption band at approximately 1738.47 cm^−1^ is related to the carboxyl group in xanthan gum, while the absorptions at approximately 1633.16–1634.91 cm^−1^ are due to C=O stretching in the pyruvate group of xanthan gum. Peaks at approximately 1400 cm^−1^ correspond to C-H stretching. Absorption bands in the 500 cm^−1^–1000 cm^−1^ range have been attributed to the stretching vibrations of =C-H, C-O, O-H, and C-C bonds [53].

^1^H NMR analysis: The ^1^H NMR results of biosynthesized xanthan gum using D-glucose and coconut palm sugar as carbon sources are shown in the Supplementary Materials (Figures S1 and S2). The signals at 1.3 ppm and 2.0 ppm were assigned to the methyl protons of the pyruvate and acetyl groups, respectively. The protons from the hydroxyl groups in xanthan gum were found around 4.0 ppm [54]. The apparent peaks at around 4.7 ppm were due to D_2_O.

## 3. Discussion

It is important to understand the production of xanthan gum by integrating key parameters for its biosynthesis using different carbon sources, fermentation duration, and the determination of antioxidant activities. It is known that glucose concentrations below 2–5% are not effective for the maximal growth of bacterial cells. On the other hand, high concentrations of glucose have no significant effect on bacterial growth as well [55].

According to the literature, the highest efficiencies have been obtained using glucose, sucrose, maltose, and starch to produce xanthan gum. For example, a yield of 13.23 g/L of xanthan gum was obtained using sucrose, 12.32 g/L maltose, 5.23 g/L fructose, 1 g/L lactose as carbon sources [40]. Polyol sugars, such as inositol and D-sorbitol, were not good carbon sources for biopolymer production. According to the literature, a yield of 1.40 g/L xanthan gum was obtained using D-sorbitol [40].

Therefore, recent studies have focused on the use of natural alternatives as substrates to produce xanthan gum. As an example, the potential of various materials as alternatives to traditional substrates for xanthan gum production was assessed. These materials included green coconut shells, passion fruit peel, straw, and corn cobs, as well as agro-industrial waste. Previous research has shown that a medium based on jackfruit seed powder is useful for the economical production of xanthan gum on low-cost substrates, thereby reducing environmental impact [56,57,58].

In this study, we investigated the relationship between the different carbon sources, their concentration, the yield of xanthan gum, and its antioxidant activity. The bioproduction of xanthan gum should also be based on the elucidation of the chemical structure of the carbon source and the biological activity relationship. The results have shown that carbon sources and renewable resources are critical components of the bacterial media for biopolymer production.

Based on the results obtained, we can conclude that after 24 h of fermentation, the highest concentrations of xanthan gum were obtained using lactose (29.19 g/L) compared to D-glucose (24.37 g/L).

After 24 h of fermentation, when analyzing the results of the study, it was found that the highest concentrations of xanthan were obtained using hydrolyzates of renewable resources, such as avocado peels (28.09 g/L), xylose (27.20 g/L), peels of lemon (26.77 g/L), as carbon sources compared to D-glucose (24.37 g/L).

After 24 h of fermentation, when analyzing the results of the study, it was found that the highest concentrations of xanthan gum were obtained using L-glutamic acid (29.61 g/L), malt extract (24.99 g/L) as nitrogen sources compared to bacterial peptone (24.37 g/L) after 24 h of fermentation. Summarizing the results of the study, it was found that the highest concentrations of xanthan gum were obtained using L-glutamic acid (32.34 g/L) and L-arginine (28.89 g/L) after 72 h of fermentation.

The highest antioxidant activities according to reducing power activity were measured for xanthan gum, whose biosynthesis was based on unrefined cane sugar, L-arabinose, and coconut palm sugar, in comparison with D-glucose.

The highest antioxidant activity according to the DPPH was obtained for xanthan gum, for which a biosynthesis of D-sorbitol (62.67%) was used as the carbon source compared to D-glucose (11.44%).

The use of cheaper and more sustainable carbon sources for xanthan gum production can reduce production costs and, therefore, improve the economic effect of the process. Future work can focus on finding new renewable substrates (apple juice residue, sugar beet pulp waste, carrot and potato peel) to produce high-value biopolymers [56].

## 4. Materials and Methods

Carbon sources were used in the production of xanthan gum. (1) D-glucose anhydrous (C_6_H_12_O_6_, Sigma-Aldrich, St. Louis, MO, USA, CAS 50-99-7) is a monosaccharide containing six carbon atoms and an aldehyde group and is, therefore, called aldohexose. (2) D-sorbitol (C_6_H_14_O_6_, Sigma-Aldrich, St. Louis, MO, USA, CAS 50-70-4) is a polyhydric alcohol with about half the sweetness of sucrose. (3) Lactose (C_12_H_22_O_11_, Sigma-Aldrich, St. Louis, MO, USA, CAS 63-42-3) is a disaccharide composed of glucose and galactose in human and cow milk. (4) Sucrose (C_12_H_22_O_11_, Sigma-Aldrich, St. Louis, MO, USA, CAS 57-50-1) is a glycosyl glycoside composed of glucose and fructose units linked by an acetal oxygen bridge from the hemiacetal of glucose to the hemiketal of fructose. It acts as an osmolyte, a sweetener, and a human metabolite. (5) D-mannitol (C_6_H_14_O_6_, Sigma-Aldrich, St. Louis, MO, USA, CAS 69-65-8) is the D-enantiomer of mannitol. (6) D-fructose (C_6_H_12_O_6_, Sigma-Aldrich, St. Louis, MO, USA, CAS 57-48-7) is a monosaccharide found in sweet fruits and honey that is soluble in water, alcohol, or ether. It is used as a preservative and in intravenous infusions for parenteral feeding. (7) Erythritol (C_4_H_10_O_4_, purchased from supermarket) is the meso-diastereomer of butane-1,2,3,4-tetrol. (8) Coconut palm sugar (purchased from supermarket) consists of sucrose (70–79%), glucose (3–9%), and fructose (3–9%). (9) L-Arabinose (C_5_H_10_O_5_, Sigma-Aldrich, St. Louis, MO, USA, CAS 5328-37-0) is a metabolite found in or produced by *Saccharomyces cerevisiae*. (10) Unrefined cane sugar consists of sucrose and a small amount (3.5 to 6.5%) of molasses. The following carbon sources were used at 20 g/L in YPD broth media instead of D-glucose: D-sorbitol, lactose, sucrose, D-mannitol, D-fructose, erythritol, and L-arabinose [59].

Nitrogen sources were used in the production of xanthan gum: (1) Casein peptone (Sigma-Aldrich, St. Louis, MO, USA, CAS 91079-40-2) is a peptone derived from casein. (2) L-glutamic acid (C_5_H_9_NO_4_, Sigma-Aldrich, St. Louis, MO, USA, CAS 56-86-0) is a proteinogenic amino acid. (3) L-arginine (C_6_H_14_N_4_O_2_, Sigma-Aldrich, St. Louis, MO, USA, CAS 74-79-3) is an L-alpha-amino acid and the L-isomer of arginine. It acts as a nutraceutical, a biomarker, and a micronutrient. (4) L-methionine (C_5_H_11_NO_2_S, Sigma-Aldrich, St. Louis, MO, USA, CAS 63-68-3) is the L-enantiomer of methionine. (5) L-tryptophan (C_11_H_12_N_2_O_2_, Sigma-Aldrich, St. Louis, MO, USA, CAS 73-22-3) is the L-enantiomer of tryptophan. It has a role as an antidepressant, a nutraceutical, a micronutrient, and a plant metabolite. (6) Malt extract (Sigma-Aldrich, St. Louis, MO, USA, CAS 8002-48-0) and (7) meat extract (Sigma-Aldrich, St. Louis, MO, USA, CAS 68990-09-0) were also used. (8) L-phenylalanine (C_9_H_11_NO_2_, Sigma-Aldrich, St. Louis, MO, USA, CAS 63-91-2) was also used, as well as (9) soy peptone (Sigma-Aldrich, St. Louis, MO, USA, CAS 91079-46-8). These sources were added to the YPD medium instead of bacterial peptone.

Xanthan gum was produced using alternative carbon sources: orange peels, tangerine peels, lemon peels, avocado peels, melon peels, apple peels, cellulose, xylose, and xylitol.

*Xanthomonas campestris* inoculation: The bacterial strain *Xanthomonas campestris*, obtained from DSMZ (DSM 1526), was used. *X. campestris* was inoculated onto Luria–Bertani (LB) agar plates. The LB agar medium consisted of peptone (10.0 g/L), yeast extract (5.0 g/L), NaCl (5.0 g/L), and agar (15.0 g/L). The plate was then incubated at 37 °C for 24 h, after which a culture colony was harvested using a sterile sampling tip. This was then transferred to a 50 mL flask containing a modified YPD broth medium consisting of bacterial peptone (25.0 g/L), yeast extract (10.0 g/L), and D-glucose (20.0 g/L). The flask was then incubated in a shaking incubator set at 37 °C at 150 rpm for 72 h. An aliquot (7 mL) of the inoculated medium was added to each 250 mL flask containing 150 mL of YPD broth. The fermentation was carried out at pH 7.0, 37 °C, and 150 rpm for 24–72 h [59].

Determination of dry bacterial biomass and xanthan gum production. The bacterial biomass and the xanthan gum were collected after 24, 48, and 72 h. To determine the biomass mass (*n* = 3), an aliquot of the medium culture of the *Xanthomonas campestris* was transferred to a pre-weighed tube. The tube was then centrifuged at 10,000× *g* for 10 min at 4 °C using a centrifuge Universal 320R (Andreas Hettich GmbH & Co. KG, Tuttlingen, Germany). After discarding the supernatant, the tube was incubated at 45 °C for 24 h in an incubator (Memmert IN55, GmbH & Co. KG, Schwabach, Germany) to dry the biomass. A solution of KCl (5 mL, at 5% *w/v* based on the final mixture) was stirred into a pre-weighed tube containing the supernatant. After adding isopropanol (35 mL), the mixture was shaken on a thermoshaker (Shaker-Incubator ES-20, Riga, Latvia) for three hours, after which the sample was refrigerated overnight. After overnight storage, the precipitated xanthan gum was centrifuged at 10,000× *g* for 10 min at 4 °C using a Universal 320R centrifuge (Andreas Hettich GmbH & Co. KG, Tuttlingen, Germany), then dried at 45 °C for 48 h in an incubator (Memmert IN55, GmbH & Co. KG, Schwabach, Germany) [59].

Production of hydrolyzates from renewable resources. The renewable resources (peels) were hydrolyzed by cellulases. The reaction was carried out in a 0.1 M citrate buffer, at pH 4.8, with a solid loading of 3% *w*/*w* and 3% *w*/*w* cellulase (CelluStar, Dyadic International, Inc., Jupiter, FL, USA) enzymes [60].

### Measurement of Antioxidant Activities

Ferric reducing antioxidant power (FRAP) assay: The FRAP reagent contained 2.5 mL of 10 mM TPTZ (2,4,6-tripyridyl-*s*-triazine) solution in 40 mM HCl, 2.5 mL of FeCl_3_ (20 mM), and 25 mL of acetate buffer (0.3 M, pH 3.6). To analyze xanthan gum samples (1 mg/mL), 50 µL were mixed with 1.5 mL of the FRAP reagent. The absorbance of the reaction mixture was measured spectrophotometrically (UV-1280 spectrophotometer (Shimadzu Corporation, Kyoto, Japan)) at 593 nm. To construct the calibration curve, five concentrations of FeSO_4_·7H_2_O (5, 10, 15, 20, and 25 μM) were used. Each experiment was repeated three times [61].

Ferric ion (Fe^3+^) reducing antioxidant power (Fe^3+^-Fe^2+^ transformation assay). Xanthan gum samples at a concentration of (1 mg/mL) 0.5 mL were mixed with phosphate buffer (1.25 mL, 0.2 M, pH 6.6) and potassium ferricyanide [K_3_Fe(CN)_6_] (1.25 mL, 1%). The mixture was then incubated at 50 °C for 20 min. Aliquots (1.25 mL) of trichloroacetic acid (10%) were then added to the mixture, which was then centrifuged for 10 min at 9000 rpm using a centrifuge Universal 320R (Andreas Hettich GmbH & Co. KG, Tuttlingen, Germany). The upper layer of the solution (1.25 mL) was mixed with distilled water (1.25 mL) and FeCl_3_ (0.25 mL, 0.1%), and the absorbance was measured at 700 nm using a UV-1280 spectrophotometer (Shimadzu Corporation, Kyoto, Japan) [61]. Each experiment was repeated three times.

1,1-Diphenyl-2-picrylhydrazyl (DPPH) radical scavenging assay: The free radical scavenging activity of xanthan gum was measured using this method. First, a 1 mg/mL solution of xanthan gum in water was prepared. Then, a 0.1 mM solution of DPPH in ethanol was prepared, and 1 mL of this solution was added to the solutions of the analyzed xanthan gum samples. The mixture was stirred vigorously and left to stand at room temperature. After 20 min, the absorbance of the reaction mixture was measured at 517 nm using a UV-1280 spectrophotometer (Shimadzu Corporation, Kyoto, Japan). Each experiment was repeated three times [62].

FT-IR spectra analysis: IR spectra (n, cm^−1^) were recorded on a Perkin–Elmer Spectrum BX FT–IR spectrometer (Perkin–Elmer Inc., Waltham, MA, USA) using KBr pellets.

^1^H NMR analysis: Proton nuclear magnetic resonance (^1^H NMR) spectra were obtained using a Bruker Avance III ^1^H 700 MHz spectrometer (Billerica, MA, USA).

Statistical analysis: The results of three experiments are expressed as the mean ± SD. Statistically significant comparisons between glucose and alternative carbon sources were tested using an unpaired two-tailed *t*-test with a significance level set at *p* < 0.1. GraphPad Prism version 8.0.2 for Windows (San Diego, CA, USA) was used for the statistical analysis.

## 5. Conclusions

Xanthan gum is an important biodegradable, biocompatible, and renewable biopolymer that is widely used in food, biotechnology, biomedical, cosmetic, food bio-packaging, and 3D printing industries [63]. Creation and development of xanthan gum with bioactive additives in composite films for the preservation of fresh-cut vegetables and fruits is one of many examples [64,65]. Xanthan gum has attracted a great deal of attention in food packaging applications due to its extreme pseudoplastic behavior and rheological properties that enhance film-forming properties [66]. One of the most sustainable solutions is to replace fossil-based polymers with biopolymers. It is also important to find alternatives to D-glucose as a carbon source in the production medium and to use renewable resources to increase the sustainability of xanthan gum production.

Our study shows that using L-glutamic acid as the carbon source for 72 h bacterial fermentation of *Xanthomonas campestris* resulted in the highest xanthan gum yield. However, a very high concentration of xanthan gum is produced in just 24 h of fermentation using hydrolyzates of avocado and lemon peels. According to the reducing power method, xanthan gum synthesized using renewable resources exhibits the highest antioxidant activity.

The results obtained are of great importance for identifying new alternative carbon sources, as well as being an attractive approach for the potential biotechnological application of biopolymers. Understanding the influence of carbon sources and various renewable resources on biopolymer production provides opportunities for future research into new biomanufacturing routes. It is estimated that the global market for xanthan gum will grow by 15% by 2027, reaching a value of approximately USD 455.9 million [67]. However, scaling up xanthan gum production from laboratory to industrial levels using new sustainable technologies and bioeconomic strategies remains challenging [68,69,70].

## Data Availability

Data is contained within the article.

## Supplementary Materials

The following supporting information can be downloaded at: https://www.mdpi.com/article/10.3390/molecules30132734/s1, Figure S1: ^1^H NMR spectra of biosynthesized xanthan gum using glucose as the carbon source; Figure S2: ^1^H NMR spectra of biosynthesized xanthan gum using coconut palm sugar as the carbon source.
