# Supplementary material for: Sustainable Production and Antioxidant Activity of Bacterial Xanthan Gum
Source: Molecules. 2025 Jun 25;30(13):2734. doi: 10.3390/molecules30132734 (PMC12250617; doi:10.3390/molecules30132734)
Supplement: Supplementary file 1 [file molecules-30-02734-s001.zip › molecules-3704699-supplementary.pdf]

Supplementary Materials

Sustainable Production and Antioxidant Activity of Bacterial Xanthan Gum

Ilona Jonuškienė [1,2,*], Erika Davicijonaitė [1], Monika Vaškevičiūtė[1], Ihsan Kala [1], Rima Stankevičienė [1], Kristina Kantminienė [3], Ingrida Tumosienė [1]

[1] Department of Organic Chemistry, Kaunas University of Technology, Radvilėnų Pl. 19,
LT-50254 Kaunas, Lithuania; erikutedavi@gmail.com (E.D.); monika.vaskeviciute99@gmail.com (M.V.); ihsan.kala13@gmail.com (I.K.); rima.stankeviciene@ktu.lt (R.S.); ingrida.tumosiene@ktu.lt (I.T.)

[2] Bioprocess Research Centre, Kaunas University of Technology, Radvilėnų Pl. 19, LT-50254 Kaunas, Lithuania

[3] Department of Physical and Inorganic Chemistry, Kaunas University of Technology, Radvilėnų Pl. 19, LT-50254 Kaunas, Lithuania; kristina.kantminiene@ktu.lt

* Correspondence: ilona.jonuskiene@ktu.lt

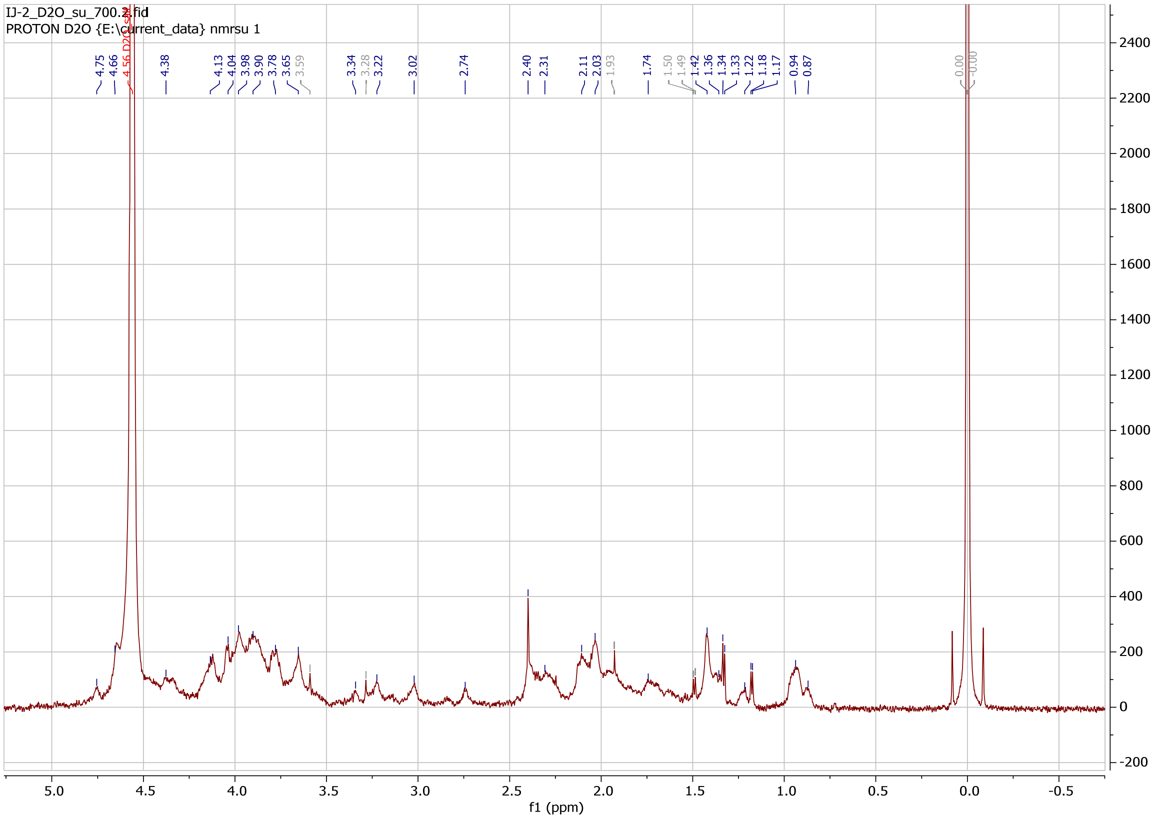

Figure S1. ^{1}H NMR spectra of biosynthesized xanthan gum using glucose as the carbon source.

The peaks appearing at about 1.3 ppm and 2.0 ppm corresponded to acetate and pyruvate groups, respectively. The hydroxyl group of xanthan gum was found around 4.0 ppm. The apparent peaks at around 4.7 ppm were due to D_2O.

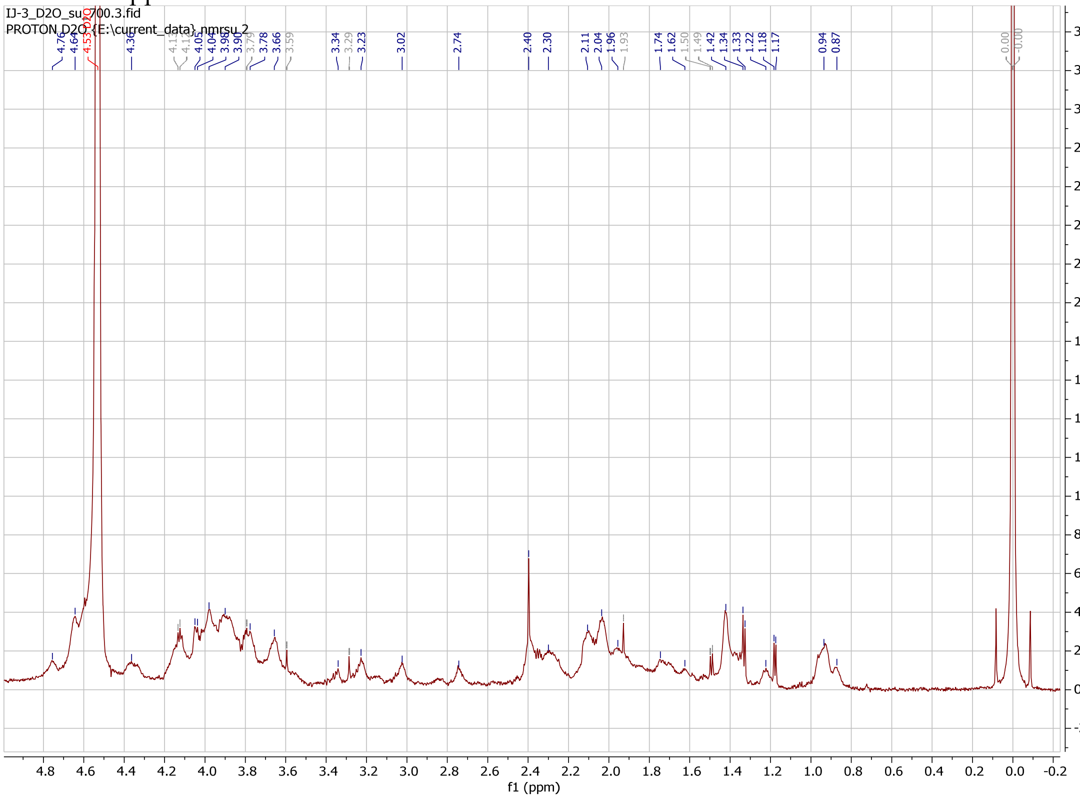

Figure S2. ^{1}H NMR spectra of biosynthesized xanthan gum using coconut palm sugar as the carbon source.

The peaks appearing at about 1.3 ppm and 2.0 ppm corresponded to acetate and pyruvate groups, respectively. The hydroxyl group of xanthan gum was found around 4.0 ppm. The apparent peaks at around 4.7 ppm were due to D_2O.
